# Supplementary material for: QTL Mapping for Important Agronomic Traits Using a Wheat55K SNP Array-Based Genetic Map in Tetraploid Wheat
Source: Plants (Basel). 2023 Feb 14;12(4):847. doi: 10.3390/plants12040847 (PMC9964379; doi:10.3390/plants12040847)
Supplement: Supplementary file 1 [file plants-12-00847-s001.zip › Supplementary figures.pdf]

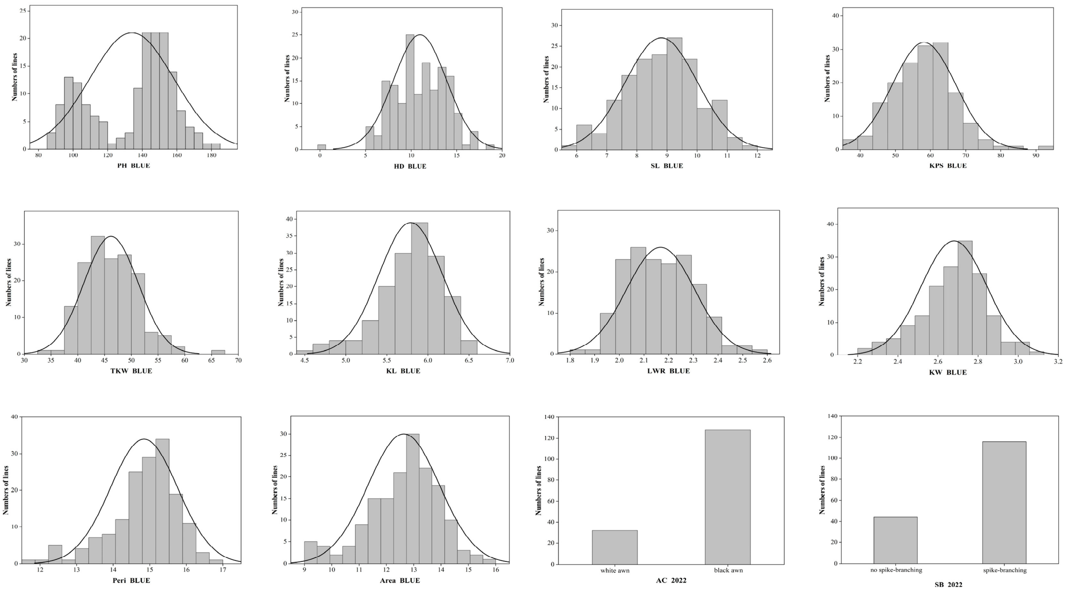

Suppl. Figure. S1 Frequency distributions for plant height (PH), heading date (HD), spike length (SL), Kernel number per spike (KPS), thousand-kernel weight (TKW), kernel length (KL), kernel width (KW), kernel length-width ratio (LWR), kernel perimeter (Peri) and kernel area (Area) based on BLUE; awn color (AC) and spike-branching (SB) in 2022.

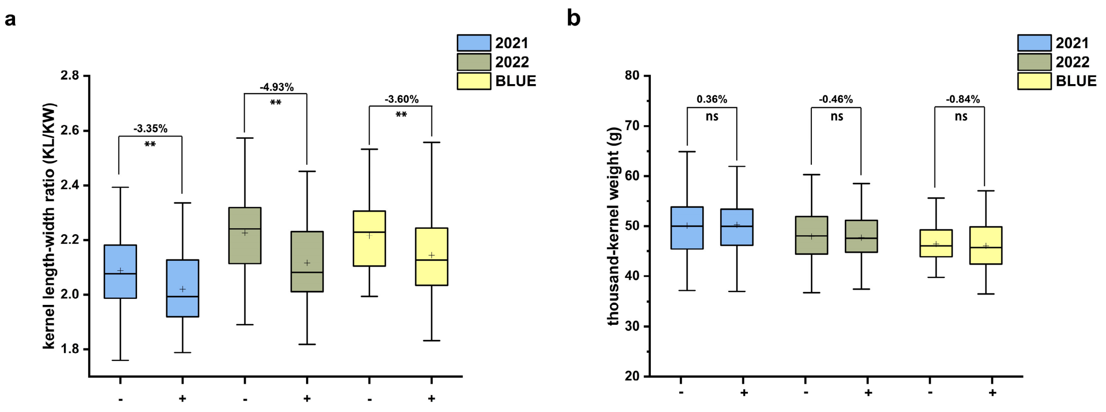

Suppl. Figure. S2 Effect of *QLWR.nwafu-7A.1* on kernel length-width ratio (LWR) (**a**) and thousand-kernel weight (TKW) (**b**). + and − represent lines with the alleles from the Icaro and Y4 of the target locus, respectively; *significant at the level of $p < 0.05$, **significant at the level of $p < 0.01$, ns: Not significant at $P > 0.05$.

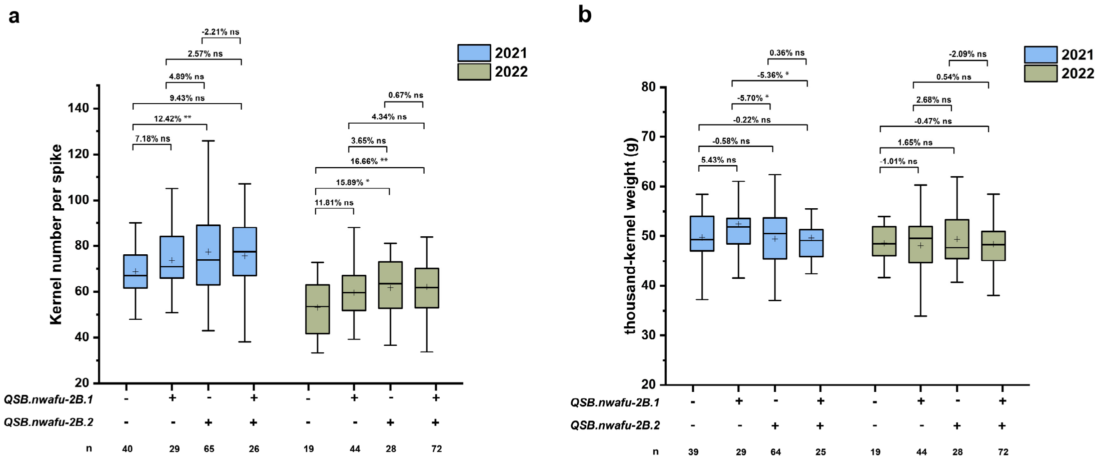

Suppl. Figure. S3 Interaction of *QSB.nwafu-2B.1* and *QSB.nwafu-2B.2* on kernel number per spike (KPS) (**a**) and thousand-kernel weight (TKW) (**b**). + and − represent lines with the alleles from the Icaro and Y4 of the target locus, respectively; * and ** Significance at P < 0.05 and P < 0.01, respectively; ns: Not significant at P > 0.05.

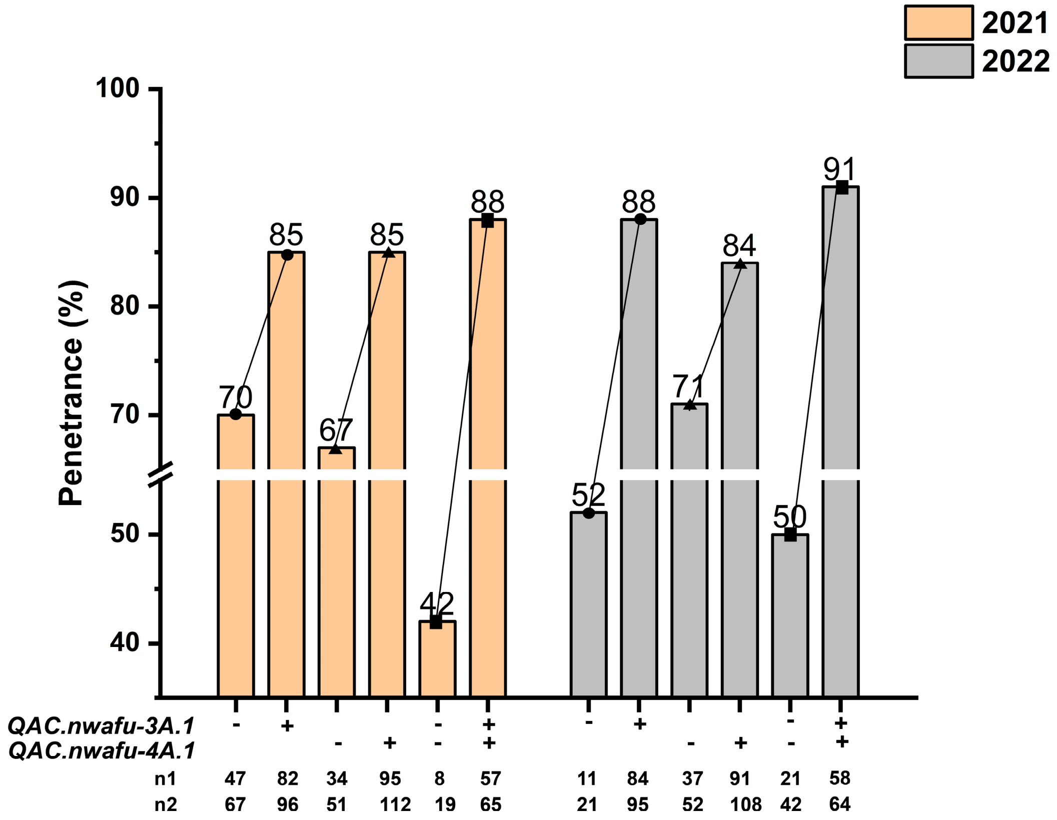

Suppl. Figure. S4 Interaction of *QAC.nwafu-3A.1* and *QAC.nwafu-4A.1* on awn color (AC). + and − represent lines with the alleles from the Icaro and Y4 of the target locus, respectively; n1 represents the number of homozygous lines containing the corresponding QTL and phenotypically exhibiting black awn, n2 represents the number of homozygous lines containing the corresponding QTL.
